# Averting the legacy of kidney disease – focus on childhood

**DOI:** 10.4102/phcfm.v8i1.1093

**Published:** 2016-04-08

**Authors:** Julie R. Ingelfinger, Kamyar Kalantar-Zadeh, Franz Schaefer

**Affiliations:** 1 World Kidney Day, International Society of Nephrology, in collaboration with the International Federation of Kidney Foundations, Brussels, Belgium

## Abstract

World Kidney Day 2016 focuses on kidney disease in childhood and the antecedents of adult kidney disease that can begin in earliest childhood. Chronic kidney disease (CKD) in childhood differs from that in adults, as the largest diagnostic group amongst children includes congenital anomalies and inherited disorders, with glomerulopathies and kidney disease in the setting of diabetes being relatively uncommon. In addition, many children with acute kidney injury will ultimately develop sequelae that may lead to hypertension and CKD in later childhood or in adult life. Children born early or who are small-for-date newborns have relatively increased risk for the development of CKD later in life. Persons with a high-risk birth and early childhood history should be watched closely to help to detect early signs of kidney disease in time to provide effective prevention or treatment. Successful therapy is feasible for advanced CKD in childhood; there is evidence that children fare better than adults, if they receive kidney replacement therapy including dialysis and transplantation, whilst only a minority of children may require this ultimate intervention. Because there are disparities in access to care, effort is needed so that children with kidney disease, wherever they live, may be treated effectively, irrespective of their geographic or economic circumstances. Our hope is that World Kidney Day will inform the general public, policymakers and caregivers about the needs and possibilities surrounding kidney disease in childhood.

‘For in every adult there dwells the child that was, and in every child there lies the adult that will be.’ (from *The Book of Lost Things*, by John Connolly)

## Introduction and overview

The eleventh World Kidney Day will be celebrated around the globe on 10 March 2016. This annual event, sponsored jointly by the International Society of Nephrology (ISN) and the International Federation of Kidney Foundations (IFKF), has become a highly successful effort to inform the general public and policymakers about the importance and ramifications of kidney disease. In 2016, World Kidney Day is dedicated to kidney disease in childhood and the antecedents of adult kidney disease, which can begin in earliest childhood.

**FIGURE 1:**
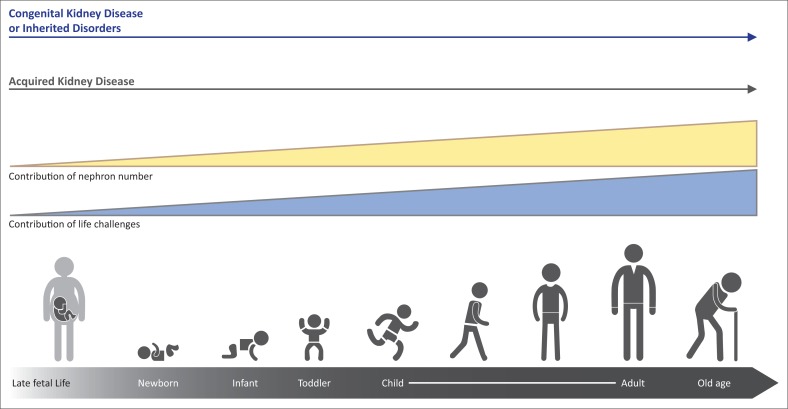
The types and risks of kidney disease change over the life cycle. The contribution of nephron number increases over the life cycle, in concert with events that provide direct insults and challenges to kidneycycle health.

Children who endure acute kidney injury (AKI) from a wide variety of conditions may have long-term sequelae that can lead to chronic kidney disease (CKD) many years later.^1,2,3,4^ Furthermore, CKD in childhood, much of it congenital, and complications from the many non-renal diseases that can affect the kidneys secondarily, not only lead to substantial morbidity and mortality during childhood but also result in medical issues beyond childhood (Figure 1). Indeed, childhood deaths from a long list of communicable diseases are inextricably linked to kidney involvement. For example, children who succumb to cholera and other diarrhoeal infections often die not from the infection but because of AKI induced by volume depletion and shock. In addition, a substantial body of data indicates that hypertension, proteinuria and CKD in adulthood have childhood antecedents – from as early as *in utero* and perinatal life (see Table 1 for definitions of childhood). World Kidney Day 2016 aims to heighten general awareness that much adult renal disease is actually initiated in childhood. Understanding high-risk diagnoses and events that occur in childhood has the potential to identify and intervene pre-emptively in those people at higher risk for CKD during their lifetimes.

**TABLE 1:** Definitions of stages of early life.

Definitions	Stages of life
Perinatal period	22 completed weeks of gestation to day 7 of postnatal life
Neonatal period	Birth to day 28 of postnatal life
Infancy	Birth to 1 year of age
Childhood	1 year of age to 10 years of age
Adolescence	10 years of age to 19 years of age

The data in this table are as defined by the World Health Organization. There is, however, variation worldwide in how these stages of early life are defined. Some would define ‘young people’ as those aged 24 years or less. In the USA, childhood as a whole is defined as up to age 21.

Worldwide epidemiological data on the spectrum of both CKD and AKI in children are currently limited, though increasing in scope. The prevalence of CKD in childhood is rare – and has been variously reported at 15.0–74.7 per million children.^3^ Such variation is likely because data on CKD are influenced by regional and cultural factors, as well as by the methodology used to generate them. The World Health Organization (WHO) has recently added kidney and urological disease to mortality information tracked worldwide, and should be a valuable source of such data over time – yet WHO does not post the information by age group.^5^ Databases such as the North American Pediatric Renal Trials and Collaborative Studies (NAPRTCS),^6^ the US Renal Data System (USRDS)^7^ and the EDTA registry^8^ include data on paediatric end-stage renal disease (ESRD), and some on CKD. Projects such as the ItalKid^9^ and Chronic Kidney Disease in Children (CKiD)^10^ studies, the Global Burden of Disease Study 2013, as well as registries that now exist in many countries, provide important information, and more is required.^11^

AKI may lead to CKD, according to selected adult population studies.^12^ The incidence of AKI amongst children admitted to an intensive care unit varies widely – from 8% to 89%.^1^ The outcome depends on the available resources. The results from projects such as the AWARE study, a five-nation study of AKI in children, are awaited.^13^ Single-centre studies as well as meta-analyses indicate that both AKI and CKD in children account for a minority of CKD worldwide.^2,3^ However, it is increasingly evident that kidney disease in adulthood often springs from a childhood legacy.

## Spectrum of paediatric kidney diseases

The conditions that account for CKD in childhood, with a predominance of congenital and hereditary disorders, differ substantially from those in adults. To date, mutations in more than 150 genes have been found to alter kidney development or specific glomerular or tubular functions.^14^ Most of these genetic disorders present during childhood, and many lead to progressive CKD. Congenital anomalies of the kidney and urinary tract (CAKUT) account for the largest category of CKD in children (Table 2) and include renal hypoplasia/dysplasia and obstructive uropathy. Important subgroups amongst the renal dysplasias are the cystic kidney diseases, which originate from genetic defects of the tubulo-epithelial cells’ primary cilia. Many paediatric glomerulopathies are caused by genetic or acquired defects of the podocytes, the unique cell type lining the glomerular capillaries. Less common but important causes of childhood CKD are inherited metabolic disorders such as hyperoxaluria and cystinosis, and atypical haemolytic uraemic syndrome, a thrombotic microangiopathy related to genetic abnormalities of complement, coagulation or metabolic pathways.

**TABLE 2:** Aetiology of chronic kidney disease in children.†

CKD aetiology	Percentage (range)	ESRD aetiology	Percentage (range)
CAKUT	48–59	CAKUT	34–43
GN	5–14	GN	15–29
HN	10–19	HN	12–22
HUS	2–6	HUS	2–6
Cystic	5–9	Cystic	6–12
Ischaemic	2–4	Ischaemic	2

Rare causes include congenital NS, metabolic diseases, cystinosis. Miscellaneous causes depend on how such entities are classified.

CAKUT, congenital anomalies of the kidney and urinary tract; GN, glomerulonephritis; HN, hypertension; HUS, haemolytic uraemic syndrome.

† from Harambat et al.^2^ CKD data are from NAPRTCS, the Italian Registry and the Belgian Registry. ESRD data are from ANZDATA, ESPN/ERA-EDTA, UK Renal Registry and the Japanese Registry.

In various classifications, it is not clear how to categorise children who have suffered AKI and apparently recovered, or how and whether to include those children who have had perinatal challenges, probably resulting in a relatively low nephron number.

Amongst children with childhood-onset ESRD, glomerulopathies are slightly more, and congenital anomalies less, common (Table 2) owing to the typically more rapid nephron loss in glomerular disease. However, recent evidence suggests that many patients with milder forms of CAKUT may progress to ESRD during adulthood, peaking in the fourth decade of life.^15^

There are national and regional differences in the type and course of both AKI and CKD during childhood and beyond. Death from kidney disease is higher in developing nations, and national and regional disparities in care and outcome must be addressed. Further, access to care is variable, depending on the region, the country and its infrastructure. By focusing on kidney disease in childhood, cost-effective solutions may be reached, as treating disease early and pre-emptively may prevent later, more advanced, CKD. Expectations depend on the availability of care and management. Treating children, even from infancy, who have AKI and CKD that requires renal replacement therapy, can be effective in mitigating the burden of kidney disease in adulthood. Doing so requires resources that focus on the most expeditious and cost-effective ways to deliver acute renal replacement therapy (RRT) in childhood.

## Congenital kidney disease and developmental origins of health and disease, renal endowment and implications

In regions where antenatal fetal ultrasounds are routine, many children with urological abnormalities are identified antenatally, which permits early intervention. However, in much of the world, children with structural abnormalities are not identified until much later, when symptoms develop. Whilst generalised screening for proteinuria, haematuria and urinary tract infections are carried out in some countries and regions, there is a lack of consensus as to its effectiveness. However, there is general agreement that children with antenatal ultrasound studies that indicate possible genito-urinary anomalies, children with a family history of kidney disease, and children with signs such as failure to thrive or a history of urinary tract infection, voiding dysfunction or an abnormal-appearing urine should be examined. Initial screening would include a focused physical examination and a urine dipstick, formal urinalysis and a basic chemistry panel, followed by a more focused evaluation if indicated.

Depending on the diagnosis, definitive therapy may be indicated. However, the evidence that therapy will slow progression of CKD in childhood remains limited. Angiotensin converting enzyme inhibitors, angiotensin receptor blockers, antioxidants and, possibly, dietary changes may be indicated, depending on the diagnosis. However, dietary changes need to permit adequate growth and development. The ESCAPE trial provided evidence that strict blood pressure control retards progression of CKD in children, irrespective of the type of underlying kidney disease.^16^

Some very young children may require RRT in early infancy. Recent data pooled from registries worldwide indicate good survival, even when dialysis is required from neonatal age.^2,17^ Kidney transplantation – the preferred RRT in children – is generally suitable after 12 months of age, with excellent patient and allograft survival, growth and development.

Evidence is accumulating that childhood-onset CKD leads to accelerated cardiovascular morbidity and shortened life expectancy. Ongoing large prospective studies such as the Cardiovascular Comorbidity in Children with CKD (4C) Study are expected to expand our knowledge of the causes and consequences of early cardiovascular disease in children with CKD.^18^

In addition to those children with congenital kidney disease, it is now known that perinatal events may affect future health in the absence of evident kidney disease in early life.^19^ Premature infants appear to be particularly at risk for kidney disease long after they are born, based both on observational cohort studies, as well as on case reports. Increasingly premature infants survive, including many born well before nephrogenesis is complete.^20^ The limited data available indicate that in the process of neonatal ICU care, such babies receive many nephrotoxins, and that those dying prior to discharge from the nursery have fewer and larger glomeruli.^21^ Additionally, those surviving have evidence of renal impairment that may be subtle.^22^ Even more concerning, abundant epidemiological data indicate that persons born at term but with relatively low birthweights may be at high risk for hypertension, albuminuria and CKD in later life.^23^ When direct measurements are pursued, such persons, as adults, may have fewer nephrons, and hence a low cardiorenal endowment.

In focusing on children for World Kidney Day, we would note that it is key to follow kidney function and blood pressure throughout life in those persons born early or small-for-date. By doing so, and avoiding nephrotoxic medications throughout life, it may be possible to avert CKD in many people.

## Resources and therapeutics for children – differences from therapeutics for adults

Disparities exist in the availability of resources to treat AKI in children and young people; consequently, too many children and young adults in developing nations succumb if AKI occurs. To address the problem, the ISN has initiated the Saving Young Lives Project, which aims both to prevent AKI with prompt treatment of infection and/or delivery of appropriate fluid and electrolyte therapy, and also to treat AKI when it occurs. This ongoing project in Sub-Saharan Africa and South-East Asia, in which four kidney foundations participate equally (IPNA, ISN, ISPD and SKCF),^†^ focuses on establishing and maintaining centres for the care of AKI, including the provision of acute peritoneal dialysis. The project links with the ISN’s 0 by 25 project, which calls on members to ensure by 2025 that nobody dies from preventable and AKI.

In view of the preponderance of congenital and hereditary disorders, therapeutic resources for children with CKD have historically been limited to a few immunological conditions. Very recently, progress in drug development in concert with advances in genetic knowledge and diagnostic capabilities has begun to overcome the long-standing ‘therapeutic nihilism’ in paediatric kidney disease. Atypical haemolytic uraemic syndrome (HUS), long considered ominous, with a high likelihood of progression to ESRD and post-transplantation recurrence, has turned into a treatable condition – with the advent of a monoclonal antibody that specifically blocks C5 activation.^24^ Another example is the use of vasopressin receptor antagonists to retard cyst growth and preserve kidney function in polycystic kidney disease.^25^ First proven efficacious in adults with autosomal dominant polycystic kidney disease, therapy with vaptans holds promise also for the recessive form of the disease, which presents and often progresses to ESRD during childhood.

However, patient benefit from pharmacological research breakthroughs is jeopardised on a global scale by the enormous cost of some of the new therapeutic agents. The quest for affordable innovative therapies for rare diseases will be a key issue in paediatric nephrology in the years to come.

The identification of children likely to benefit from novel therapeutic approaches will be greatly facilitated by the development of clinical registries that help to clarify the natural disease course, including genotype-phenotype correlations. Apart from disease-specific databases, there is also a need for treatment-specific registries. These are particularly relevant in areas where clinical trials are difficult to perform owing to small patient numbers and lack of industry interest, as well as for therapies in need of global development or improvement. For instance, there is currently a large international gradient in the penetration and performance of paediatric dialysis and transplantation. Whereas paediatric patient and technique survival rates are excellent and even superior to those of adults in many industrialised countries, it is estimated that almost half of the world’s childhood population is not offered chronic renal replacement therapy (RRT) at all. Providing access to RRT for all children will be a tremendous future challenge. To obtain reliable information on the demographics and outcomes of paediatric RRT, the International Pediatric Nephrology Association (IPNA) is about to launch a global population-based registry. If successful, the IPNA RRT registry might become a role model for global data collection.

## Transition from paediatric to adult care

Transition of care for adolescents with kidney disease into an adult setting is critical both for patients and their caregivers. Non-adherence is a too-frequent hallmark of transition from paediatric to adult care for young patients with chronic disease states.^26,27,28^ Consequently, considered steps combined with systematically defined procedures supported by validated pathways and credible guidelines must be in place to ensure successful outcomes.

In the process of change from paediatric to adult care, ‘transition’, which should occur gradually, must be distinguished from ‘transfer’, which is often an abrupt and mechanistic change in provider setting. Introducing the concept of transition should be pre-emptive, starting months to years prior to the targeted time, as children move into adolescence and adulthood. The ultimate goal is to foster a strong relationship and individualised plan in the new setting that allows the patient to feel comfortable enough to report non-adherence and other lapses in care.

A transition plan must recognise that the emotional maturity of children with kidney disease may differ widely. Assessment of the caregiver and the family structure as well as cultural, social and financial factors at the time of transition are most important, including a realistic assessment of caregiver burden.^4^ The appropriate timing and format of transition may vary widely amongst different patients and in different settings; therefore, a flexible process without a set date and even without a delineated format may be preferable.

Importantly, transition may need to be slowed, paused or even reversed temporarily during crises such as disease flares or progression, or if family or societal instability occurs. A recent joint consensus statement by the ISN and IPNA proposed steps consistent with the points just outlined, aiming to enhance the transition of care in kidney disease in clinical practice.^29,30^

## Call for generating further information and action

Given the vulnerabilities of children with kidney disease, including the impact on growth and development and future life as an adult, and given the much greater proportion of children in developing nations facing resource constraints, educating everyone involved is imperative to realign communications and actions.^31,32^ These efforts should foster regional and international collaborations and exchange of ideas between local kidney foundations, professional societies, other not-for-profit organisations, and states and governments, to empower all stakeholders to improve the health, well-being and quality of life of children with kidney diseases and to ensure their longevity into adulthood.

Until recently, however, the WHO consensus statement on non-communicable diseases (NCD) included cardiovascular disease, cancer, diabetes and chronic respiratory disease, but not kidney disease.^33,34^ Fortunately, and owing in part to a global campaign led by the ISN, the Political Declaration on NCDs from the United Nations Summit in 2011 mentioned kidney disease under Item 19.^35^

Increasing education and awareness about renal diseases in general and kidney disease in childhood in particular is consistent with the objectives of the WHO to reduce mortality from NCD with a 10-year target population level, initiatives focusing on changes in lifestyle (including tobacco use reduction, salt intake control, dietary energy control, and alcohol intake reduction) and effective interventions (including blood pressure, cholesterol and glycaemic control). Heightened efforts are needed to realign and expand these multidisciplinary collaborations with more effective focus on early detection and management of kidney disease in children. Whereas the issues related to kidney disease may be overshadowed by other NCDs with apparently larger public health implications such as diabetes, cancer, and cardiovascular diseases, our efforts should also increase education and awareness on such overlapping conditions as cardiorenal connections, the global nature of CKD and ESRD as major NCDs, and the role of kidney disease as the multiplier disease and confounder for other NCDs. White papers including consensus articles and blueprint reviews by world-class experts can serve to enhance these goals.^36^

## Footnotes

† The four partners are (in alphabetical order): IPNA (International Pediatric Nephrology Association), ISN (International Society of Nephrology), ISPD (International Society for Peritoneal Dialysis), SKCF (Sustainable Kidney Care Foundation).
