# Corrigendum: Averting the legacy of kidney disease – focus on childhood

**DOI:** 10.4102/phcfm.v9i1.1706

**Published:** 2017-12-13

**Authors:** Julie R. Ingelfinger, Kamyar Kalantar-Zadeh, Franz Schaefer

**Affiliations:** 1 World Kidney Day, International Society of Nephrology, in collaboration with the International Federation of Kidney Foundations, Brussels

In the version of this article initially published, the abbreviation HN located in the footnote for Table 2 was identified incorrectly as Hypertension. The correct definition for HN is Hereditary Nephropathy.

This correction does not alter the study’s findings of significance or overall interpretation of the study results. The errors have been corrected in the PDF version of the article. The authors apologise for any inconvenience caused.

